# Predictors of high-acuity outcomes among 10–17-year-olds utilizing pediatric emergency services in Taiwan: a sex-based comparison of male and female adolescents

**DOI:** 10.1186/s12873-025-01237-5

**Published:** 2025-05-16

**Authors:** Mei-Wen Wang, Jiun-Hau Huang

**Affiliations:** 1 Department of Family Medicine, Chang Gung Memorial Hospital, Keelung Branch, Keelung, Taiwan; 2 Department of Family Medicine, Chang Gung Memorial Hospital, Linkou Branch, Taoyuan, Taiwan; 3 Institute of Health Policy and Management, College of Public Health, National Taiwan University, Taipei, Taiwan; 4 Institute of Health Behaviors and Community Sciences, College of Public Health, National Taiwan University, 17 Xu-Zhou Road, Taipei, 10055 Taiwan; 5 Department of Public Health, College of Public Health, National Taiwan University, Taipei, Taiwan

**Keywords:** Adolescents, Emergency medicine, Emergency department, High-acuity outcomes, Predictors, Chief complaints, Sex differences, Taiwan

## Abstract

**Background:**

Adolescents’ emergency department (ED) presentations are multi-faceted. However, patient characteristics and chief complaints associated with severe outcomes, particularly sex differences, remain underexplored. This study aimed to identify predictors of high-acuity outcomes among adolescents utilizing pediatric emergency services in Taiwan and to examine sex differences.

**Methods:**

We analyzed data from 16,910 non-traumatic pediatric ED visits by adolescents aged 10–17 years at a major tertiary-care hospital in northern Taiwan (2009–2014). Trauma-related cases were not included, as they follow distinct clinical pathways. We tracked four severe outcomes longitudinally as high-acuity outcomes and used them in predictive modeling: (1) intensive care unit (ICU) admission or in-ED death; (2) inpatient ward admission; (3) return to the ED within 72 h for the same presenting complaint; and (4) ED length of stay exceeding 6 h. We used chi-square (*χ*²) and Fisher’s exact tests to analyze bivariate associations. Multivariate logistic regression models, stratified by sex, were constructed with final model selection based on the lowest Akaike Information Criterion (AIC) value to optimize model fit and parsimony. Results are presented as adjusted odds ratios (AORs) and 95% confidence intervals (CIs).

**Results:**

A total of 2,508 adolescents (14.8%) experienced at least one high-acuity outcome. In the final model for all adolescents, the following patient characteristics were significantly associated with high-acuity outcomes: male sex (AOR = 0.90, 95% CI: 0.82–0.98); ages 16–17 (AOR = 1.23, 95% CI: 1.10–1.37); triage levels 1–2 (AORs = 1.98–2.27, 95% CIs: 1.45–3.00), indicating greater urgency for intervention; ≥2 abnormal vital signs (AORs = 1.59–1.91, 95% CIs: 1.08–2.87); and a Glasgow Coma Scale score of 13–14 (AOR = 0.49, 95% CI: 0.26–0.94), indicating mild impairment of consciousness. In this overall model, we also identified 10 chief complaints as significant predictors of high-acuity outcomes, including endocrine-related diseases (AOR = 2.10, 95% CI: 1.52–2.91), skin-related diseases (AOR = 1.95, 95% CI: 1.02–3.73), nervous system diseases (AOR = 1.34, 95% CI: 1.08–1.68), and poisoning (AOR = 1.38, 95% CI: 1.06–1.81). Among male adolescents, the significant chief complaints mirrored those in the overall model, except that eye diseases (AOR = 1.47, 95% CI: 1.01–2.17) emerged as an additional male-only predictor, and headaches were not, but musculoskeletal system diseases (AOR = 1.45, 95% CI: 1.01–2.08) were retained in the male-specific model. By contrast, only two chief complaints remained significant predictors among female adolescents: endocrine-related diseases (AOR = 1.97, 95% CI: 1.31–2.98) and headaches (AOR = 0.72, 95% CI: 0.54–0.96).

**Conclusions:**

This study demonstrated that male and female adolescents with high-acuity outcomes exhibited distinct clinical profiles, underscoring the importance of sex-specific approaches in pediatric emergency care. Our empirical findings highlight the need for heightened clinical attention to adolescents presenting with certain chief complaints. By identifying predictors of high-acuity outcomes, this study contributes to improving clinical decision-making and quality assessment in ED settings. These findings may also inform preventive strategies and early interventions in broader healthcare contexts, including school-based and primary care services.

**Clinical trial number:**

Not applicable.

## Introduction

Adolescents have distinct health service needs because of the unique challenges of this developmental stage. In contrast to children and adults, adolescents undergo rapid physical, intellectual, and emotional growth, making their health a critical priority for healthcare systems [1]. Historically, clinical attention has focused on outpatient settings or school health services; however, the complexity of adolescent presentations in the emergency department (ED) necessitates greater focus [1, 2]. ED presentations among adolescents are often more multi-faceted than those of adults, and their conditions can be harder to assess because of variability in clinical manifestations [3].

Numerous factors, such as mental health and psychosocial issues, influence adolescent ED presentations [4, 5]. Age and sex are particularly important determinants of ED outcomes, affecting the severity and acuity of clinical conditions [6]. Understanding the predictors of high-acuity outcomes in pediatric emergency services is critical for improving adolescent health and preventing severe complications.

Pediatric EDs in Taiwan are separate from general medical EDs and cater exclusively to patients under the age of 18. Previous research has highlighted the differences in medical performance and care methods between children and adults [7]. In the ED, young children often present with less severe conditions than adults [7, 8]. Additionally, the chief complaints and likelihood of hospital admission vary with age in pediatric EDs [9, 10]. Adolescents frequently exhibit different levels of disease severity and clinical complaints, compared with adults. Understanding why adolescents visit the ED and identifying true medical emergencies is crucial, but the literature provides limited insight into ED outcomes for this population.

In Taiwan, the National Health Insurance (NHI) system and subsidized medical services reduce the financial burden of ED visits for children and adolescents, which may encourage families to seek emergency care even for non-acute conditions. Previous studies have shown that adolescents often visit the ED for chronic or recurrent complaints rather than acute emergencies [5]. Mental health and psychosocial conditions are increasingly common reasons for ED visits among adolescents, with age and sex playing a significant role in shaping these presentations [4, 11]. As such, accurate and swift diagnosis is essential, particularly for complex presentations requiring objective criteria to assess acuity.

The 5-level triage system, such as the commonly used Emergency Severity Index (ESI), is a critical component of ED operations, providing a standardized and rapid assessment of patient urgency. The triage system is widely recognized for its strong predictive power in clinical assessment. However, it is essential to recognize that although the triage system offers a valuable initial evaluation, it represents a snapshot of the patient’s condition at arrival. While effective for initial categorization, the triage system alone may not fully capture the dynamic evolution of patient acuity. Disease progression and individual patient characteristics can lead to instances where the initial triage score does not accurately reflect the patient’s evolving condition, sometimes resulting in under-triaged cases with severe outcomes. For instance, some patients with seemingly low triage scores may subsequently require critical care admission [12]. Therefore, to comprehensively assess patient acuity and enhance clinical protocols and triage practices, it is crucial to consider a wider range of severity indicators.

To account for the evolution of patient acuity beyond the initial triage assessment, we should include other markers of severity to better understand the predictors that reflect the broader spectrum of high-acuity presentations in adolescent emergency care. Specifically, prior research has shown that the following indicators significantly impact adolescent health and offer valuable insights into patient outcomes: intensive care unit (ICU) admission, mortality, inpatient ward admission, extended ED length of stay, and repeated ED visits for the same complaints [3, 8, 10, 13]. These markers provide a more comprehensive view of patient acuity, addressing a gap left by previous research that primarily concentrated on extreme events, such as out-of-hospital cardiac arrest (OHCA) [10].

Furthermore, recognizing the need for a more comprehensive assessment of adolescent ED patients, we should draw from both subjective and objective information gathered during ED visits, in addition to utilizing the standard 5-level triage classification system. For instance, previous research has identified factors associated with better outcomes among children, but these findings are not specific to adolescents [14, 15]. Moreover, most prior studies have focused on the psychological conditions of adolescents without incorporating objective clinical data and presentations [4]. This highlights the crucial need for a comprehensive approach that includes both clinical factors and objective manifestations when assessing adolescent ED outcomes. Therefore, our study specifically focuses on patients’ subjective chief complaints, encompassing mental disorders, along with other objective patient characteristics, such as the number of abnormal vital signs.

### The current study

Given the gap in existing research on high-acuity risk among ED-visiting adolescents in Taiwan, this study aimed to identify predictors of high-acuity outcomes among adolescents utilizing pediatric emergency services in Taiwan, and explore whether these predictors varied between male and female patients. Notably, we focused exclusively on non-traumatic pediatric ED visits, rather than trauma-related ED visits, which follow distinct clinical pathways. These findings are expected to inform proactive and preemptive interventions to mitigate the risk of severe outcomes and to help differentiate patients who most need pediatric ED services. Furthermore, by identifying sex-specific risk factors, this study could guide the development of tailored strategies to prevent or reduce repeated ED visits, ultimately improving the quality of healthcare for adolescents.

## Methods

### Participants, study setting, and data source

The data for this study were extracted from the pediatric ED records of a major tertiary-care hospital in northern Taiwan from 2009 to 2014. The dataset initially included 17,239 ED visits by adolescent patients aged 10 to 17 years who presented with non-traumatic conditions (i.e., medical illnesses, infections, and non-injury-related complaints).

To ensure that our analysis remains focused on identifying factors related to high-acuity outcomes among adolescents presenting with acute, non-traumatic conditions, we excluded cases with:

1. Terminal illnesses: These patients require frequent medical care, often for palliative purposes, and their inclusion could confound the analysis of acute medical presentations.
2. Chronic diseases: Chronic conditions necessitate ongoing medical management, and their inclusion might obscure factors specifically associated with high-acuity outcomes in acute settings.
3. Cognitive impairments equivalent to those in patients younger than 10 years: Severe cognitive impairments may limit symptom reporting and influence triage decisions.
4. Traumatic seizure disorders: These cases follow distinct clinical pathways and were not the focus of this study.
5. Recent-onset seizure illnesses: Seizure presentations often require separate diagnostic and treatment considerations.
6. Out-of-hospital cardiac arrest (OHCA): These patients represent a unique emergency population, typically requiring immediate resuscitation rather than assessment of non-traumatic chief complaints.

In addition, we also excluded cases with any missing data for any of the six key patient characteristics (sex, age group, triage level, time of the ED visit, number of abnormal vital signs, and Glasgow Coma Scale score) or any of the 20 chief complaints examined in this study. This strict exclusion criterion for missing data was implemented to ensure that the analyzed records reflected actual data collected at the adolescent ED visit, rather than extrapolated or imputed data.

### Measures

#### High-acuity outcomes

This study longitudinally tracked five outcomes following adolescent ED visits to create a composite high-acuity outcome variable. Four of these outcomes were categorized as high-acuity: (1) intensive care unit (ICU) admission or in-ED death; (2) inpatient ward admission; (3) return to the ED within 72 h for the same presenting complaint (a factor associated with increased mortality rates) [16]; and (4) ED length of stay exceeding 6 h (associated with prolonged hospital stays and increased mortality) [17]. The fifth outcome represented patients discharged from the ED post-treatment without experiencing any of the four high-acuity events. These outcomes were chosen because of their clinical relevance in assessing patient severity.

A dichotomous outcome variable was created for logistic regression analysis, indicating the occurrence or absence of any high-acuity outcome. If any of the four high-acuity outcomes occurred, it was coded as 1 (event). If none of the four occurred (i.e., the patient was discharged without experiencing any high-acuity events), it was coded as 0 (reference group). Given the exploratory nature of our study and the relatively low frequency of individual high-acuity events, this composite outcome variable allowed us to assess the predictors of a range of severe outcomes.

#### Age group

Age was categorized into three groups: 10–12, 13–15, and 16–17 years. This categorization aligns with the key educational stages in the Taiwanese school system—elementary, junior high, and senior high school, respectively.

#### Triage level

The 5-level triage classification system is used in Taiwan. Level 1 represents the most critical cases requiring immediate life-saving interventions, whereas level 5 represents non-urgent conditions. Triage-level data were systematically gathered from the ED triage sheets for each patient.

#### Time of the ED visit

The visiting times were categorized into three groups based on the working shifts of the medical staff: 08:00–15:59, 16:00–23:59, and 00:00–07:59. These intervals also align with the typical school hours and rest periods of the adolescent patients.

#### Number of abnormal vital signs

Vital signs, including heart rate, blood pressure, body temperature, and respiratory rate, were classified as normal or abnormal according to the age-specific reference ranges established by the Ministry of Health and Welfare in Taiwan. A vital sign was considered abnormal if it fell outside these predefined age-specific ranges. The number of abnormal vital signs was then tallied for each patient. Participants were categorized into four groups according to the count of their abnormal vital signs: 0, 1, 2, and ≥ 3.

#### Glasgow coma scale

The Glasgow Coma Scale (GCS) [18, 19] was used to assess consciousness levels in patients with acute conditions. It evaluates eye-opening, verbal, and motor responses separately, and summates the scores, ranging from 3 (deep coma or death) to 15 (fully awake). Lower scores indicate deeper unconsciousness and more severe injury. Brain injury severity is generally categorized as: severe (GCS ≤ 8), moderate (GCS 9–12), and mild (GCS 13–15). Thus, this study categorized GCS into four groups: 15, 13–14, 9–12, and 3–8.

#### Chief complaints

Chief complaints were categorized using the standard ICD-9 and ICD-10 classification systems. We then compiled a list of 20 categories using the available data within our pediatric ED records, specifically excluding those for cancer and unknown organ infectious diseases. Specifically, cancer diagnoses were excluded because they often involve chronic and specialized care, which may not accurately reflect the patterns of acute, non-traumatic ED visits in adolescents. Similarly, unknown organ infectious diseases were excluded as they can be complex and require specialized diagnostic and treatment approaches, potentially introducing variability that could confound the analysis of more common chief complaints. Instead, common symptoms, such as chest pain, headaches, abdominal pain, and hyperventilation syndrome, were included. The complete list of these 20 categories is detailed in Table 1.

**Table 1:** Chief complaints and their bivariate associations with high-acuity outcomes among 10–17-year-old adolescents utilizing pediatric emergency services in Taiwan, by sex

Chief Complaint	Total		High-Acuity Outcome^a^								
			All			Male			Female		
	*n*	Col %	*n*_HAO_	Row %	*P*	*n*_HAO_	Row %	*P*	*n*_HAO_	Row %	*P*
Endocrine, nutritional, and metabolic diseases, and immunity disorders	37	0.2	26	70.3	***	5	31.3		21	100	***
Diseases of the blood and blood-forming organs	2	0.01	1	50.0		0	0		1	100	
Mental disorders	91	0.5	29	31.9	***	13	28.3	*	16	35.6	***
Diseases of the nervous system	576	3.4	155	26.9	***	92	30.3	***	63	23.2	***
Eye diseases	485	2.9	12	2.5	***	5	2.0	***	7	3.1	***
Ear diseases	225	1.3	10	4.4	***	6	4.8	***	4	4.0	***
Diseases of the circulatory system	68	0.4	12	17.7		5	18.5		7	17.1	
Diseases of the respiratory system	2,624	15.5	286	10.9	***	181	11.4	***	105	10.1	***
Diseases of the digestive system	1,365	8.1	278	20.4	***	133	17.7	**	145	23.7	***
Diseases of the genitourinary system	174	1.0	30	17.2		16	18.4		14	16.1	
Complications of pregnancy, childbirth, and puerperium^b^	7	0.1^b^	3	42.9^b^		-	-	-	3	42.9	
Diseases of the skin and subcutaneous tissue	1,144	6.8	105	9.2	***	43	6.4	***	62	13.1	
Diseases of the musculoskeletal system and connective tissue	325	1.9	42	13.0		19	11.2		23	14.7	
Fever	4,913	29.1	718	14.6		382	13.8		336	15.6	
Chest pain	293	1.7	51	17.4		41	20.9	**	10	10.3	
Headache	723	4.3	59	8.2	***	32	8.4	***	27	7.9	***
Abdominal pain	3,356	19.8	624	18.9	***	324	19.1	***	300	18.1	***
Hyperventilation syndrome	40	0.2	3	7.5		0	0		3	10.0	
Poisoning	11	0.1	8	72.7	***	6	66.7	***	2	100	*
Others that are hard to classify	451	2.7	56	12.4		35	13.4		21	11.1	

^a^ The four high-acuity outcomes include: (1) intensive care unit (ICU) admission or in-ED death; (2) inpatient ward admission; (3) return to the ED within 72 h for the same presenting complaint; and (4) ED length of stay exceeding 6 h. Col % represents the percentage of ED visits attributed to each chief complaint, calculated as (*n*/Total ED visits) × 100. It reflects the proportion of ED visits for a specific chief complaint relative to all ED visits included in the study, providing an estimate of the prevalence of that complaint among the total ED visits. *n*_*HAO*_ represents the number of ED visits with the chief complaint listed in that row that resulted in any high-acuity outcome. Row % indicates the percentage of ED visits with that chief complaint that resulted in any high-acuity outcome, calculated as (*n*_*HAO*_/*n*) x 100. The “Total” column’s *n* represents the total number of ED visits for that chief complaint and is the denominator for the “All” Row %. The *n* values for “Male” and “Female” are not shown but can be calculated using their respective Row % values. For each row: *n* (Total) = *n* (Male) + *n* (Female); *n*_*HAO*_ (All) = *n*_*HAO*_ (Male) + *n*_*HAO*_ (Female)

^b^ Only for female adolescents

*P < 0.05, **P ≤ 0.01, ***P ≤ 0.001

### Procedures and statistical analyses

Univariate odds ratios were first estimated to examine associations between the six key patient characteristics and high-acuity outcomes. Bivariate analyses were conducted using chi-square (*χ*²) and Fisher’s exact tests, as appropriate, to assess the associations of sample characteristics and chief complaints with high-acuity outcomes. Chi-square tests were used for categorical variables, with Fisher’s exact test applied when any expected cell count was below five to maintain statistical validity.

Multivariate logistic regression analyses were then conducted to estimate the adjusted odds of high-acuity outcomes associated with patient characteristics and chief complaints. Logistic regression was used, given the binary nature of the outcome variable. To ensure appropriate statistical adjustment in the multivariate models, six key patient characteristics (sex, age group, triage level, time of the ED visit, number of abnormal vital signs, and Glasgow Coma Scale score) were selected a priori, considering their clinical relevance to ED outcomes and their potential confounding effects on the study results. Accordingly, these six variables were included as covariates in all multivariate models, except for sex in the sex-stratified models.

Given the exploratory aims of the study, we applied a backward selection procedure based on Akaike Information Criterion (AIC) values to identify the best-fitting model. The model with the lowest AIC value was selected as the final model, as this approach balances model fit and complexity by penalizing overfitting. Unlike *P*-value-based selection procedures, the AIC-based approach focuses on optimizing overall model performance and mitigates the risk of Type I error associated with multiple comparisons. Although some predictors retained in the final model did not reach statistical significance at the conventional *α* = 0.05 level, they were retained, considering their contribution to the model’s overall parsimony and fit.

Additionally, we independently assessed multicollinearity among predictors to ensure robust model estimation. As our models were developed for exploratory purposes rather than for use in a predictive context, we did not perform external validation or assess predictive performance measures (e.g., area under the curve). Adjusted odds ratios (AORs) and 95% confidence intervals (CIs) are reported for all final models.

Although beyond the primary scope of this study, we conducted an ancillary analysis to explore which individual high-acuity outcomes primarily contributed to the composite outcome. Specifically, for the chief complaints retained in the final model for all adolescents, we estimated univariate ORs and 95% CIs for each high-acuity outcome individually. This analysis focused on predictors retained in the final model to enhance interpretability and preserve statistical power. A column was added to the final table to present the outcome-specific ORs and 95% CIs; intervals that did not cross 1.00 were interpreted as evidence of an association between the predictor and the corresponding outcome.

This study adheres to the Strengthening the Reporting of Observational Studies in Epidemiology (STROBE) guidelines for observational research. All statistical analyses were conducted using SPSS and SAS.

## Results

Of the 17,239 adolescent ED visits initially identified, 129 cases were excluded according to the pre-defined clinical exclusion criteria, and an additional 200 cases were excluded owing to missing data for any of the key patient characteristics or chief complaints. After these exclusions, the final analytic sample comprised 16,910 ED visits. Details and rationale for the exclusion criteria, as well as variable selection, are provided in the Methods section.

Table 2 presents the sample characteristics and univariate odds ratios associated with high-acuity outcomes. In total, 2,508 (14.8%) of the 16,910 pediatric ED visits resulted in high-acuity events. Males constituted 55.3% of the sample. The majority were classified as triage level 3 (75.3%). Most ED visits occurred between 16:00–23:59 (46.5%), whereas 33.4% took place during daytime hours (08:00–15:59). Regarding the number of abnormal vital signs, 31.3% exhibited none, 34.7% one, 25.6% two, and 8.4% three or more. Most had a GCS score of 15 (96.8%). In Table 2, univariate ORs for all six key patient characteristics had 95% CIs that did not cross 1.00, indicating an association with high-acuity outcomes. Table 3 presents the sex-stratified bivariate associations between patient characteristics and high-acuity outcomes. All five patient characteristics exhibited significant results, except for age in males.

**Table 2:** Sample characteristics and univariate odds ratios associated with high-acuity outcomes among 10–17-year-old adolescents utilizing pediatric emergency services in Taiwan

Variable	Total (*N* = 16,910)		High-acuity outcome^a^ (*N* = 2,508)		
	*n*	Col %	*n*_HAO_	Row %	OR (95% CI)
**Sex**					
Female	7,555	44.7	1,170	15.5	Ref
Male	9,355	55.3	1,338	14.3	0.91 (0.84–0.99)
**Age Group (years)**					
10–12	6,622	39.2	930	14.0	Ref
13–15	6,199	36.7	907	14.6	1.05 (0.95–1.16)
16–17	4,089	24.2	671	16.4	1.20 (1.08–1.34)
**Triage Level** ^b^					
Level 1	426	2.5	91	21.4	Ref
Level 2	1,217	7.2	222	18.2	0.82 (0.63–1.08)
Level 3	12,726	75.3	1,969	15.5	0.67 (0.53–0.85)
Level 4	2,499	14.8	225	9.0	0.36 (0.28–0.48)
Level 5	42	0.3	1	2.4	0.09 (0.01–0.66)
**Time of the ED Visit**					
08:00–15:59	5,646	33.4	941	16.7	Ref
16:00–23:59	7,856	46.5	1,051	13.4	0.77 (0.70–0.85)
00:00–07:59	3,408	20.2	516	15.1	0.89 (0.79–1.00)
**Number of Abnormal Vital Signs** ^c^					
0	5,284	31.3	617	11.7	Ref
1	5,868	34.7	920	15.7	1.41 (1.26–1.57)
2	4,332	25.6	751	17.3	1.59 (1.41–1.78)
≥ 3	1,426	8.4	220	15.4	1.38 (1.17–1.63)
**Glasgow Coma Scale** ^d^					
15	16,373	96.8	2,363	14.4	Ref
13–14	408	2.4	74	18.1	1.31 (1.02–1.70)
9–12	116	0.7	61	52.6	6.58 (4.56–9.49)
3–8	13	0.1	10	76.9	19.76 (5.44–71.86)

^a^ The four high-acuity outcomes include: (1) intensive care unit (ICU) admission or in-ED death; (2) inpatient ward admission; (3) return to the ED within 72 h for the same presenting complaint; and (4) ED length of stay exceeding 6 h. Col % represents the proportion of cases within each category of the variable, calculated as (*n* for the category/Total *N*) × 100. The percentages are specific to each variable and sum to 100% within each variable because all cases are distributed across the defined categories. *n*_*HAO*_ represents the number of ED visits listed in that row that resulted in any high-acuity outcome. Row % indicates thepercentage of ED visits listed in that row that resulted in any high-acuity outcome, calculated as (*n*_*HAO*_/*n*) x 100. OR: univariate odds ratio; Ref: Reference

^b^ Level 1 indicates the most urgent condition that requires immediate treatment. Level 5 indicates the least urgent condition

^c^ Vital signs include: heart rate, blood pressure, body temperature, and respiratory rate

^d^ A neurological scale consisting of three tests: eye, verbal, and motor responses, with a score of 3 indicating the most severe condition

**Table 3:** Sample characteristics and their bivariate associations with high-acuity outcomes among 10–17-year-old adolescents utilizing pediatric emergency services in Taiwan, by sex

Variable	Male					Female				
	Total (*N* = 9,355)		High-acuity outcome^a^ (*N* = 1,338)			Total (*N* = 7,555)		High-acuity outcome^a^ (*N* = 1,170)		
	*n*	Col %	*n*_HAO_	Row %	*P*	*n*	Col %	*n*_HAO_	Row %	*P*
**Age Group (years)**										*
10–12	3,807	40.7	529	13.9		2,815	37.3	401	14.2	
13–15	3,471	37.1	478	13.8		2,728	36.1	429	15.7	
16–17	2,077	22.2	331	15.9		2,012	26.6	340	16.9	
**Triage Level** ^b^					*					*
Level 1	233	2.5	50	21.5		193	2.6	41	21.2	
Level 2	686	7.3	124	18.1		531	7.0	98	18.5	
Level 3	7,045	75.3	1,053	14.9		5,681	75.2	916	16.1	
Level 4	1,364	14.6	111	8.1		1,135	15.0	114	10	
Level 5	27	0.3	0	0		15	0.2	1	6.7	
**Time of the ED Visit**					*					*
08:00–15:59	3,059	32.7	494	16.1		2,587	34.2	447	17.3	
16:00–23:59	4,374	46.8	574	13.1		3,482	46.1	477	13.7	
00:00–07:59	1,922	20.5	270	14.0		1,486	19.7	246	16.6	
**Number of Abnormal Vital Signs** ^c^					*					*
0	2,710	29.0	314	11.6		2,574	34.1	303	25.9	
1	3,370	36.0	494	14.7		2,498	33.1	426	36.4	
2	2,467	26.4	416	16.9		1,865	24.7	335	28.6	
≥3	808	8.6	114	14.1		618	8.2	106	9.1	
**Glasgow Coma Scale** ^d^					*					*
15	9,034	96.6	1,261	14.0		7,339	97.1	1,102	15.0	
13–14	246	2.6	39	15.9		162	2.1	35	21.6	
9–12	63	0.7	29	46.0		53	0.7	32	60.4	
3–8	12	0.1	9	75.0		1	0.0	1	100.0	

^a^ The four high-acuity outcomes include: (1) intensive care unit (ICU) admission or in-ED death; (2) inpatient ward admission; (3) return to the ED within 72 h for the same presenting complaint; and (4) ED length of stay exceeding 6 h. Col % represents the proportion of cases within each category of the variable, calculated as (*n* for the category/Total *N*) × 100. The percentages are specific to each variable and sum to 100% within each variable because all cases are distributed across the defined categories. *n*_*HAO*_ represents the number of ED visits listed in that row that resulted in any high-acuity outcome. Row % indicates the percentage of ED visits listed in that row that resulted in any high-acuity outcome, calculated as (*n*_*HAO*_/*n*) x 100

^b^ Level 1 indicates the most urgent condition that requires immediate treatment. Level 5 indicates the least urgent condition

^c^ Vital signs include: heart rate, blood pressure, body temperature, and respiratory rate

^d^ A neurological scale consisting of three tests: eye, verbal, and motor responses, with a score of 3 indicating the most severe condition

*P < 0.05

Table 1 presents the 20 chief complaints, their frequency distributions, and the proportions of ED visits resulting in high-acuity outcomes, stratified by sex. Overall, the five most common chief complaints were fever (29.1%), abdominal pain (19.8%), diseases of the respiratory system (15.5%), diseases of the digestive system (8.1%), and diseases of the skin and subcutaneous tissue (6.8%). Of these five, only fever was not significantly associated with high-acuity outcomes. Poisoning and endocrine-related diseases had the highest proportions of high-acuity outcomes (72.7% and 70.3%, respectively).

Table 4 presents three multivariate logistic regression models for predictors of high-acuity outcomes for all, male, and female adolescents, respectively. In the overall model, the following patient characteristics exhibited significant associations with high-acuity outcomes: male sex (AOR = 0.90, 95% CI: 0.82–0.98), ages 16–17 years (AOR = 1.23, 95% CI: 1.10–1.37), triage levels 1–2 (AORs = 1.98–2.27, 95% CIs: 1.45–3.00), two or more abnormal vital signs (AORs = 1.59–1.91, 95% CIs: 1.08–2.87), and GCS score 13–14 (AOR = 0.49, 95% CI: 0.26–0.94). In addition, chief complaints pertaining to endocrine-related disorders (AOR = 2.10, 95% CI: 1.52–2.91) exhibited the strongest association with high-acuity outcomes, whereas headaches (AOR = 0.74, 95% CI: 0.58–0.95) were negatively associated with high-acuity outcomes (Table 4).

**Table 4:** Multivariate logistic regression models for predictors of high-acuity outcomes among 10–17-year-old adolescents utilizing pediatric emergency services in Taiwan, by sex

Variable	All	Male	Female
	AOR (95% CI)	AOR (95% CI)	AOR (95% CI)
**Sex**			
Female	Ref		
Male	0.90 (0.82–0.98)		
**Age Group (years)**			
10–12	Ref	Ref	Ref
13–15	1.04 (0.94–1.15)	1.01 (0.88–1.15)	1.10 (0.93–1.30)
16–17	1.23 (1.10–1.37)	1.26 (1.09–1.46)	1.18 (0.99–1.42)
**Triage Level**			
Level 1	2.27 (1.71–3.00)	2.51 (1.70–3.71)	1.94 (1.21–3.10)
Level 2	1.98 (1.45–2.71)	2.15 (1.47–3.15)	1.84 (1.20–2.83)
Level 3	1.24 (0.93–1.66)	1.36 (0.94–1.96)	1.10 (0.73–1.66)
Level 4	Ref	Ref	Ref
Level 5	0.75 (0.41–1.35)	0.82 (0.36–1.85)	0.68 (0.26–1.76)
**Time of the ED Visit**			
08:00–15:59	0.90 (0.77–1.06)	0.92 (0.74–1.15)	0.88 (0.68–1.14)
16:00–23:59	0.93 (0.79–1.10)	0.96 (0.77–1.19)	0.89 (0.68–1.16)
00:00–07:59	Ref	Ref	Ref
**Number of Abnormal Vital Signs**			
0	Ref	Ref	Ref
1	1.21 (0.89–1.64)	1.29 (0.84–1.97)	1.14 (0.72–1.81)
2	1.59 (1.08–2.34)	1.67 (1.03–2.73)	1.51 (0.84–2.71)
≥3	1.91 (1.27–2.87)	2.12 (1.27–3.52)	1.74 (0.95–3.19)
**Glasgow Coma Scale**			
15	Ref	Ref	Ref
13–14	0.49 (0.26–0.94)	0.52 (0.21–1.27)	0.44 (0.18–1.10)
9–12	1.15 (0.40–3.30)	1.29 (0.31–5.33)	0.94 (0.16–5.64)
3–8	3.41 (0.77–15.15)	4.89 (0.68—35.32)	2.56 (0.33–19.66)

Variable	All		Male	Female		
	AOR (95% CI)	Outcome-specific OR (95% CI)	AOR (95% CI)		AOR (95% CI)	
**Chief Complaints**						
Endocrine, nutritional, and metabolic diseases, and immunity disorders	2.10 (1.52–2.91)	(1) 227.63 (110.14–470.43) (2) 4.04 (1.90–8.58)	2.27 (1.46–3.55)		1.97 (1.31–2.98)	
Diseases of the nervous system	1.34 (1.08–1.68)	(1) 2.64 (1.05–6.62) (2) 1.54 (1.21–1.96) (4) 1.35 (1.05–1.73)	1.43 (1.05–1.93)		1.30 (0.97–1.74)	
Eye diseases	--	--	1.47 (1.01–2.17)		--	
Ear diseases	1.50 (1.02–2.21)	(1) 3.73 (1.25–11.10) (2) 1.53 (1.10–2.13)	1.62 (1.01–2.58)		1.44 (0.86–2.41)	
Diseases of the circulatory system	1.56 (1.14–2.15)	(1) 2.65 (1.03–6.85) (2) 1.46 (1.06–2.00)	1.67 (1.13–2.47)		1.50 (0.99–2.28)	
Diseases of the genitourinary system	1.29 (1.01–1.66)	--	1.35 (1.01–1.82)		1.25 (0.89–1.74)	
Diseases of the skin and subcutaneous tissue	1.95 (1.02–3.73)	(1) 5.44 (1.16–25.62) (2) 2.05 (1.07–3.93)	2.08 (1.02–4.24)		1.78 (0.75–4.27)	
Diseases of the musculoskeletal system and connective tissue	1.32 (1.01–1.73)	(2) 1.35 (1.02–1.78)	1.45 (1.01–2.08)		--	
Fever	1.28 (1.01–1.62)	--	1.30 (0.93–1.81)		1.33 (0.93–1.89)	
Headache	0.74 (0.58–0.95)	(2) 0.69 (0.52–0.91)	--		0.72 (0.54–0.96)	
Poisoning	1.38 (1.06–1.81)	(2) 1.42 (1.07–1.89)	1.48 (1.04–2.10)		1.36 (0.94–1.96)	
**Akaike Information Criterion (AIC) Value**	10,961.46		6,091.34		4,855.94	

Note. The four high-acuity outcomes include: (1) intensive care unit (ICU) admission or in-ED death; (2) inpatient ward admission; (3) return to the ED within 72 h for the same presenting complaint; and (4) ED length of stay exceeding 6 h. The dichotomous outcome variable in these logistic regression models indicates the occurrence of any high-acuity outcome, with patients discharged without a high-acuity event serving as the reference group. “--“ indicates that the variable is not included in the AIC-based final model

AOR: adjusted odds ratio; CI: confidence interval; OR: unadjusted univariate odds ratio corresponding to a specific high-acuity outcome, as denoted by (1), (2), (3), or (4); Ref: reference

In the sex-specific models, only endocrine-related disorders were significantly associated with high-acuity outcomes among both male (AOR = 2.27, 95% CI: 1.46–3.55) and female (AOR = 1.97, 95% CI: 1.31–2.98) adolescents. The chief complaints retained in the male-specific model were the same as those in the overall model, except that an additional chief complaint related to eye diseases (AOR = 1.47, 95% CI: 1.01–2.17) was added, and headaches were removed. By contrast, female adolescents had fewer sex-specific predictors of high-acuity outcomes than their male counterparts. The only significant chief complaint other than endocrine-related disorders in the female-specific model was headaches (AOR = 0.72, 95% CI: 0.54–0.96).

Multicollinearity was assessed for these three final models, and all variance inflation factors (VIFs) were well below 10, indicating no substantial concern. Given that all predictors were dummy-coded categorical variables, the risk of multicollinearity was minimal. This confirms that these predictor variables retained in the AIC-based models were independent and suitable for inclusion in the multivariate models.

## Discussion

This study extends the existing literature by examining and comparing the patient characteristics and chief complaints that predicted high-acuity outcomes among male and female adolescents aged 10–17 years utilizing pediatric emergency services in Taiwan. Our multivariate analyses revealed that male adolescents utilizing ED services were less likely to experience high-acuity outcomes than their female counterparts. In addition to sex differences and varying sex-specific chief complaints, several patient characteristics—including age, triage level, number of abnormal vital signs, and GCS score—predicted high-acuity outcomes among adolescents.

### Older age as a predictor of high-acuity outcomes in adolescents

Our study observed that older adolescents, particularly those aged 16–17, exhibited significantly increased odds of high-acuity outcomes, compared with their younger counterparts aged 10–12. Notably, this association remained significant in the male-specific model but became marginally non-significant among female adolescents after adjusting for other covariates in the final model. Overall, this finding aligns with existing literature indicating that older adolescents may be more susceptible to severe health events necessitating post-ED hospitalization. For instance, the U.S. national ED survey data indicated that 1.6% of ED visits by those aged 15–24 were classified in the highest category of hospitalization rate (over 20%), whereas only 0.2% of ED visits by those aged 0–14 were in this category [20]. This finding also suggests that younger adolescents may visit the ED for reasons beyond immediate medical emergencies, including psychosocial concerns or barriers to primary care access. Thus, emergency care providers should exercise heightened vigilance when assessing older adolescents, potentially requiring more aggressive monitoring and management strategies.

#### Clinical implications for practice and further research

Identifying age as a predictor of high-acuity outcomes has several clinical implications. First, triage protocols should incorporate age as a risk factor, with older adolescents receiving prioritized assessment and monitoring. This may involve more comprehensive evaluations and proactive management strategies tailored to this age group. Second, the observed sex-specific differences warrant further investigation. The marginally non-significant finding in females after adjusting for other covariates suggests that high-acuity outcomes in female adolescents may be more strongly influenced by patient characteristics and chief complaints other than age, in contrast to their male counterparts. Future research should investigate these sex-based differences to refine risk stratification models.

Further studies could also explore whether social determinants—such as access to care, health literacy, or psychosocial stressors—contribute to the observed disparities. Additionally, understanding the specific medical conditions driving higher acuity in older males could help tailor interventions aimed at early identification and prevention. Lastly, integrating these findings into clinical guidelines and physician training programs can enhance adolescent ED assessments, ensuring sex- and age-specific considerations are incorporated into triage and management strategies.

### Triage levels as key predictors of high-acuity outcomes in adolescents

Our findings indicate that adolescent patients assigned triage level 1 had the highest risk for high-acuity outcomes, followed by those categorized as levels 2 and 3, compared with level 4 patients as the reference group (who typically require medical attention within one hour). These results affirm the overall effectiveness of the current ED triage system in identifying high-risk patients while highlighting the need for greater differentiation between levels 2 and 3, given the overlap in their 95% CIs and the non-significant result observed for level 3.

Previous research in adult populations has reported that 23.7% of adults were classified in the highest urgency categories (triage levels 1 and 2, requiring treatment within 15 min) and 64.7% required attention within one hour [7, 8, 13]. International studies have shown that while ED triage systems effectively stratify patient acuity, triage level distribution differs between adult and adolescent cohorts [21], with adult cases generally triaged more accurately than pediatric ones [22]. In our study, only 2.5% and 7.2% of adolescents were classified as triage levels 1 and 2, respectively, whereas a substantial proportion (75.3% and 14.8%) were designated as levels 3 and 4, corresponding to treatment within 30 min and one hour.

Notably, even those assigned to triage level 3 in our study exhibited an elevated high-acuity risk, although the AOR in the final model was marginally non-significant. Therefore, despite the lower proportions of adolescents in the highest urgency categories compared with adults, our results underscore the clinical relevance of these triage levels in adolescents. This finding is consistent with studies conducted in other countries, such as Canada, which have shown that higher triage levels are associated with increased risk of hospitalization and critical care admission in pediatric ED [23].

#### Clinical implications for practice and further research

Our findings have significant clinical implications. First, the validation of the existing triage system supports its continued use; however, the observed overlap in high-acuity risk estimates between levels 2 and 3 suggests that integrating additional clinical criteria or diagnostic tools could further enhance triage precision. Second, understanding these risk profiles can inform ED resource allocation and staff training to ensure that adolescent patients receive timely care. Finally, regarding further research and implementation, our results highlight the need for future longitudinal studies and interventional research to assess whether modified triage protocols can improve patient outcomes. Moreover, the implementation of age-specific risk scoring systems within the ED triage process may enhance accuracy. Additionally, educational programs for ED staff should emphasize the unique characteristics of adolescent patients and the importance of accurate triage assessment.

### Abnormal vital signs and high-acuity risk: Sex-based variations in adolescents

In this study, approximately 70% of adolescent ED visits involved at least one abnormal vital sign. Adolescents with 2 or ≥ 3 abnormal vital signs exhibited significantly higher risks of high-acuity outcomes, suggesting a threshold of 2 abnormal vital signs for heightened risk. Notably, the association between the number of abnormal vital signs and high-acuity risk was not significant among female adolescents, warranting further investigation.

Overall, our findings align with international studies demonstrating that abnormal vital signs are strong predictors of adverse outcomes in pediatric ED populations, such as hospital admission and intensive care unit stays [24]. The recognition of abnormal vital signs as a key indicator of patient deterioration is a cornerstone of emergency medicine practice globally [25]. However, while abnormal vital signs provide objective indicators, age-specific standards must be considered. Additionally, the potential influence of underlying mental health conditions on vital sign abnormalities in adolescents should be taken into account. In our study, the non-significant effect of two or more abnormal vital signs in female adolescents, after adjusting for other chief complaints such as nervous system diseases, supports this consideration.

#### Clinical implications for practice and further research

The above findings from this study have important clinical implications. First, they reinforce the prognostic value of abnormal vital signs in adolescent ED assessment. Given the observed significant risk elevation, ED protocols should consider using two abnormal vital signs as a clinically meaningful threshold for heightened monitoring and early intervention. Second, our results suggest that sex-specific variations in high-acuity risk merit further exploration. The non-significant finding of abnormal vital signs among female adolescents after adjusting for other chief complaints suggests that psychological factors and associated distress may partially contribute to abnormal vital signs in this group. As such, future studies could investigate whether integrating mental health screening into ED assessments improves risk prediction and patient outcomes. Finally, further research could explore whether a dose-response relationship exists between abnormal vital signs and high-acuity outcomes, which could enhance triage precision.

### Glasgow coma scale and high-acuity risk: an unexpected pattern in adolescents

In this study, over 99% of adolescent ED patients had a Glasgow Coma Scale (GCS) score between 13 and 15, indicating no severe impairment of consciousness. The GCS remains a widely used objective tool for assessing neurological function through visual, verbal, and motor responses. Surprisingly, our final model showed that adolescents scoring 13–14 (mild impairment) exhibited a significantly lower risk of high-acuity outcomes, compared with those scoring 15 (no impairment), whereas more severe impairment (scoring 9–12 and 3–8) did not correspond with significantly elevated risk.

Several reasons may explain the above finding. First, adolescents may have greater neurological resilience compared with adults, allowing them to compensate for mild impairment without leading to severe deterioration. Second, patients with moderate-to-severe GCS impairment might receive immediate intensive monitoring or interventions, mitigating their high-acuity risk and preventing adverse outcomes. Third, in pediatric populations, a lower GCS score may reflect transient rather than critical conditions, meaning it does not always indicate severe illness. For example, transient conditions like intoxication, concussion, or anxiety-related responses could momentarily lower GCS scores without posing serious risks. Lastly, given that only a small proportion of adolescents had low GCS scores (≤ 12), the lack of statistical significance might reflect sample size limitations rather than a true absence of risk.

Despite this unexpected pattern in adolescents, the GCS remains a well-established tool for predicting outcomes across various patient populations [26]. Globally, the GCS serves as a fundamental tool for assessing neurological status in emergency settings. Studies performed outside of Taiwan have also demonstrated the predictive power of the GCS regarding patient outcomes, especially in those with traumatic brain injuries [27]. However, it is important to note that the GCS should always be used in conjunction with other clinical findings, as it is only one component of a comprehensive assessment.

#### Clinical implications for practice and further research

The aforementioned findings have critical implications for ED practice. First, they support refining triage protocols to better stratify adolescents with mild-to-moderate GCS impairment. Given the significantly lower risk among those scoring 13–14, ED clinicians should consider reassessing triage criteria to ensure that adolescents in this category receive appropriate monitoring without unnecessary resource allocation. This could involve refining risk stratification frameworks to distinguish true neurological compromise from transient or non-critical impairments. Second, future research could examine which specific GCS components—visual, verbal, or motor—are most predictive of adverse outcomes in adolescent patients. Understanding these distinctions could improve risk stratification and enhance the accuracy of triage systems. Additionally, further research could explore whether integrating supplementary clinical markers, such as additional neurological assessments or patient-reported symptoms, can improve predictive accuracy in this subgroup. Lastly, studies incorporating biomarkers or neuroimaging findings alongside GCS scores may help refine prognostic models for adolescent patients presenting with altered consciousness.

### Adolescent-specific chief complaints as predictors of high-acuity outcomes

Our study revealed distinct patterns of chief complaints associated with high-acuity outcomes in adolescents, compared with adults. Previous research in adult patients over 18 (triage levels 3–5) identified nausea, vomiting, and diarrhea as predictors of critical admission [12]. By contrast, our study found that these chief complaints related to the digestive system were not significantly associated with high-acuity outcomes in 10–17-year-olds. Furthermore, aligning with pediatric studies highlighting immune diseases, unstable mental status, poisoning, and respiratory conditions as risk factors [3, 10], our research also identified endocrine and immunity diseases, nervous system diseases, and poisoning as significant predictors of high-acuity outcomes in adolescents.

In our final multivariate models, chief complaints regarding endocrine and immunity disorders had the largest effect sizes. While common adolescent complaints such as fever remained critical indicators of high-acuity risk, neurological conditions (excluding headaches) also emerged as significant predictors of high-acuity outcomes. These findings highlight the potential for increased prevalence of psychiatric and neurological issues among adolescents, aligning with the global recognition of mental health issues (including suicidal ideation, self-harm, and substance use disorders) as a significant concern in the adolescent population presenting to EDs [28]. This underscores the necessity for comprehensive mental health assessments in both school settings and during ED visits.

#### Clinical implications for practice and further research

The clinical implications of the above findings are multifaceted. First, pediatric ED practice should recognize the distinct presentation of high-acuity risk in adolescents compared with adults. While common adult predictors such as gastrointestinal complaints may not be significant in this age group, adolescent-specific indicators, such as endocrine and immunity disorders, neurological conditions (beyond headaches), and poisoning, warrant heightened attention. Second, the strong association of endocrine and immunity complaints, alongside neurological issues potentially reflecting the increased prevalence of mental health concerns in this population, underscores the critical need for comprehensive mental health assessments during adolescent ED visits, in addition to thorough evaluations of physical symptoms. Also, implementing routine mental health screenings and ensuring the availability of behavioral health professionals can facilitate early identification and intervention, potentially preventing progression to high-acuity conditions. These findings can inform the development of adolescent-specific risk stratification tools and clinical pathways within the pediatric ED setting.

Further research could explore the underlying mechanisms driving these age-related differences in high-acuity outcomes. Longitudinal studies examining the progression of specific chief complaints to severe outcomes can provide deeper insights, informing targeted interventions. Additionally, evaluating the effectiveness of integrated mental health services in EDs on patient outcomes could substantiate the benefits of such models in pediatric emergency care.

### Sex-based considerations in female adolescent ED acuity

Our study revealed sex-based differences in predictors of high-acuity outcomes among adolescents seeking ED care in Taiwan. Notably, female adolescents presenting with (1) endocrine, nutritional, and immunity disorders, (2) blood and blood-forming organ diseases, or (3) poisoning exhibited a 100% rate of high-acuity outcomes in bivariate analyses. Although the latter two conditions only had a single-digit number of cases in this study and thus did not remain significant predictors in the multivariate final model, this finding underscores critical clinical implications and suggests a potential vulnerability among female adolescents in Taiwan, warranting further investigation.

While studies specifically focusing on these three chief complaints in adolescent ED visits with sex-based comparisons are limited, existing research provides relevant context. For example, studies on endocrine disorders, such as type 1 diabetes, demonstrate that adolescent females may experience more severe complications and poorer glycemic management, compared with males, potentially owing to hormonal fluctuations and psychosocial factors [29]. Similarly, research on hematologic disorders, such as iron deficiency anemia, highlights the increased risk in adolescent females due to menstruation and nutritional deficiencies [30]. Poisoning, particularly intentional overdoses, is also a significant concern in adolescent females, often linked to underlying mental health conditions [31]. These international studies support the concept that specific biological and psychosocial vulnerabilities exist within female adolescents, potentially contributing to increased ED presentation acuity.

#### Clinical implications for practice and further research

The aforementioned findings have important clinical implications. First, EDs should consider implementing sex-specific protocols for these conditions, ensuring rapid endocrinology and immunology consults and thorough hematologic evaluations. For poisoning, systematic psychosocial assessments may be warranted, necessitating integrated mental health services within the ED. Recognizing the heightened risk, staff should be trained to identify subtle decompensation signs and suicidal ideation. Additionally, while pregnancy-related complaints were not statistically significant in this study, pregnancy in young adolescents often coincides with social and psychological challenges, such as limited access to prenatal care, an increased risk of complications, and mental health issues [32]. Therefore, clinicians must remain vigilant for associated psychosocial vulnerabilities, ensuring appropriate referrals. These findings necessitate targeted training for ED staff, emphasizing female adolescent-specific risk factors. Further research should focus on underlying mechanisms and intervention effectiveness, ultimately improving ED care for this vulnerable population.

### Sex-based chief complaints in male adolescent ED acuity

Our final model identified poisoning as a significant predictor of high-acuity outcomes among male adolescents. This finding is consistent with the upward trend revealed in a review of intentional suspected-suicide self-poisoning cases reported to the National Poison Data System from U.S. poison centers, which concluded that the incidence of self-poisoning in youth under 19 and the severity of outcomes both increased significantly after 2011, although such cases occurred predominantly in young girls [33]. In our study, however, most poisoning cases occurred in male adolescents, which could be attributable to cultural differences in substance use and access, or variations in healthcare-seeking behaviors. Another possible explanation is that adolescent males are more likely than females to present with substance-related issues often involving alcohol, which exacerbates drug poisoning. For instance, data from the U.S. Nationwide Inpatient Sample revealed significant increases in co-occurring alcohol overdoses in drug poisoning cases among adolescents ages 12–17, which further increased the severity of drug poisoning and hospitalization rates [34].

Diseases of the circulatory system also emerged as a male-specific predictor of high-acuity ED outcomes. This aligns with prior research showing that myocarditis or pericarditis tends to occur more frequently in adolescent males than females [35]. This sex-based difference may partly explain the heightened acuity, longer ED stays, or higher hospitalization rates, as evidenced among adolescents presenting to the ED with cardiogenic chest discomfort [36]. In addition, our study identified eye, ear, and skin diseases as male-specific high-acuity predictors. However, these findings are in direct contrast to the results of an analysis based on U.S. national ED survey data, indicating that ED visits for eye and ear concerns, as well as skin rashes, had an extremely low risk for hospitalization (< 1%) in the age groups 0–14 and 15–24 [20]. This cross-national divergence warrants further investigation to explore potential causes of such differences.

Additionally, musculoskeletal and genitourinary system complaints were significantly associated with high-acuity outcomes only in the male-specific model. Male adolescents generally have greater muscle mass and bone density, which can lead to different injury patterns compared with females, and they tend to engage in more high-impact sports and physical activities, which may lead to more severe musculoskeletal outcomes at ED visits [37]. While less studied in this context, genitourinary complaints could indicate underlying urinary tract infections or other serious conditions, such as testicular torsion, which often require urgent intervention.

#### Clinical implications for practice and further research

Clinically, these findings underscore the need for sex-specific strategies in pediatric emergency care. First, regarding poisoning, hospitals should consider integrating toxicology screening and risk assessment of substance use and mental health concerns in any male adolescents presenting with altered mental status or suspected ingestion. Given these risks, targeted initiatives, such as school-based substance use education and mental health interventions, are crucial for preventing severe poisoning cases in this demographic. Second, for potential cardiogenic chest pain, clinicians should maintain a high index of suspicion for serious underlying conditions, even in the absence of typical cardiac symptoms during male adolescent ED visits. This could include the use of ECGs, biomarker testing, and other diagnostic tools to ensure timely identification and management of severe circulatory system issues. Third, for eye, ear, skin, and musculoskeletal complaints, heightened clinical awareness is essential, particularly in male adolescents. Enhanced screening and early intervention strategies may facilitate the timely management of severe conditions. Fourth, for genitourinary complaints, prompt evaluation and appropriate testing for infections or other serious conditions are crucial. Improved triage protocols for these sex-specific complaints in males could potentially reduce morbidity and long-term complications.

Future research should explore the underlying behavioral and physiological mechanisms contributing to these sex-based differences and evaluate the effectiveness of intervention strategies in reducing high-acuity ED outcomes among male adolescents.

### Lower-acuity chief complaint: a potential “false alarm” in female adolescent ED visits

Our multivariate final models identified a chief complaint associated with a significantly lower risk of high-acuity outcomes (AOR < 1.00), suggesting a potential “false alarm”. Specifically, presentations related to headaches predicted lower acuity risk among female adolescents. This finding that headaches predict lower acuity aligns with international studies. While headaches are a frequent neurological complaint in adolescent ED visits [38], the majority are often attributed to benign causes, such as upper respiratory tract infections with fever, sinusitis, or migraine, and life-threatening causes are relatively rare [39]. The headache complaint may also be related to cultural perceptions of illness. However, beyond primary medical causes, it is crucial to recognize that many adolescents with psychiatric illnesses initially present with somatic symptoms, including headaches and abdominal pain, rather than overt psychiatric complaints [40]. Studies indicate that pediatric patients with underlying anxiety disorders and depression frequently report these physical symptoms in ED settings [11].

#### Clinical implications for practice and further research

These findings have significant clinical implications. First, while headaches are generally not critical, the link between somatic complaints and potential underlying psychiatric illnesses underscores the need for routine mental health screening in adolescent ED visits to enhance early detection and intervention, even when presenting complaints appear benign. This could lead to better mental health support and reduce unnecessary ED visits, improving resource allocation. Second, this female-specific lower-acuity predictor can inform revised triage protocols to prevent unnecessary ED resource utilization, allowing for quicker triage and reducing wait times for more critical cases. Implementing protocols that expedite the management of related chief complaints could also improve patient flow and overall ED efficiency.

Future studies could investigate the long-term outcomes of female adolescents presenting with this lower-acuity complaint and evaluate the effectiveness of targeted interventions for this population. Studies that include a larger sample size and a broader spectrum of mental health evaluations would be beneficial. Further research could explore strategies for differentiating benign versus high-risk presentations of common complaints, particularly in adolescents with frequent ED visits, to improve care efficiency and outcomes. Our study findings could also be incorporated into physician training programs and clinical guidelines to enhance the assessment and management of adolescent ED patients.

### Study limitations and future directions

This study has several limitations that should be acknowledged and addressed in future research and practice. First, there is a potential risk of selection bias due to reliance on data from a tertiary-care hospital. However, to gain perspective, it is essential to consider relevant factors within Taiwan’s healthcare context, which may help mitigate this concern. For instance, our NHI system plays a crucial role in shaping ED utilization patterns, as it minimizes financial barriers and provides relatively affordable access to emergency care. This affordability allows patients to self-select EDs on the basis of non-medical factors, such as geographic proximity and perceived convenience, regardless of the hospital’s level of care. Moreover, our focus on non-traumatic ED visits adds complexity. Unlike EMS-transported patients who undergo pre-screening and are directed to appropriate facilities, walk-in patients may choose EDs according to personal preference rather than clinical necessity.

As such, it remains unclear whether the hospital’s level of care reflects the severity of patients visiting the ED in our study, and how this affects generalizability. Given the NHI program, it is arguable that selection bias based on tertiary-care level is minimized, but not eliminated. Future research should investigate differences in chief complaints, post-ED outcomes, and high-acuity predictors between EMS-transported and walk-in patients. Additionally, comparative studies across primary, secondary, and tertiary care EDs would provide valuable insights.

Second, the use of a composite outcome measure represents a limitation. We acknowledge that the included components (ICU admission/death, ward admission, 72-hour return visit, and ED stay > 6 h) represent varying degrees of severity. However, given the exploratory nature of this study and the relatively low frequency of individual high-acuity events, we opted for a composite outcome to ensure sufficient statistical power for our model estimations. We recognize that this approach limits our ability to identify predictors specific to each outcome component. Future research could benefit from stratified analyses of individual high-acuity outcomes to identify outcome-specific predictors.

Finally, the observation that over 85% of adolescents in this study were discharged home without further medical intervention raises concerns about potential resource inefficiencies and generalizability. The NHI’s financial accessibility may contribute to patients overestimating their need for emergency care, treating the ED as a convenient option rather than a resource for true emergencies. This pattern of usage requires attention from policymakers to ensure appropriate utilization of emergency services. Given the tertiary-care setting and the high percentage of discharged patients, it is possible that this dataset contains a mix of both severe and less severe cases. This may limit generalizability to primary or secondary care EDs, where patient profiles and utilization patterns likely differ. In resource-limited settings, future research should explore the causal relationships between sex-specific acuity risk patterns, chief complaints, and patient outcomes. These findings could inform strategies for optimizing ED resource allocation and improving triage systems, ultimately enhancing medical efficiency and patient prognosis.

## Conclusions

In conclusion, this study provides critical insights into the predictors of high-acuity outcomes in the ED among adolescents aged 10–17, a transitional age group distinct from both younger children and adults. We revealed significant sex-based differences and highlighted the importance of a comprehensive clinical evaluation. Our findings underscore that age, triage level, abnormal vital signs, GCS score, and key adolescent-specific chief complaints, such as endocrine-related disorders, nervous system diseases, skin-related diseases, and poisoning, are crucial determinants of patient outcomes.

Specifically, in resource-limited settings, our findings can directly inform clinical protocols and triage practices. For example, the observed association between higher triage levels (1–2) and increased risk of high-acuity outcomes suggests that even in environments with limited resources, prioritizing patients based on triage scores is essential. Implementing rapid assessment protocols for these higher-risk patients, even with limited diagnostic tools, can significantly improve outcomes. Furthermore, the strong predictive value of abnormal vital signs indicates that basic vital sign monitoring should be a cornerstone of triage, regardless of resource availability. In settings where advanced diagnostics are scarce, simple tools like pulse oximetry and manual blood pressure measurement can provide crucial information for risk stratification.

The identification of nervous system diseases and poisoning as significant adolescent-specific predictors of high-acuity outcomes emphasizes the need for streamlined mental health screening and toxicology risk assessment, even in resource-constrained EDs. Utilizing brief, validated screening tools and establishing clear referral pathways to mental health professionals may enhance early intervention.

Moreover, the observed sex-based differences necessitate tailored clinical approaches. For male adolescents presenting with musculoskeletal or genitourinary complaints, even in settings with limited diagnostic capacity, clinicians should prioritize focused evaluations and consider urgent referrals. For female adolescents with endocrine-related disorders or suspected poisoning, rapid access to specialist consultation, even via telemedicine in remote areas, is crucial.

While our study provides valuable insights into the predictors of high-acuity outcomes, future longitudinal studies are essential to explore the causal relationships between the identified risk factors and high-acuity outcomes in adolescent ED patients. Such research could help refine clinical decision-making protocols, enhance triage systems, and optimize resource allocation, ultimately leading to improved patient care and outcomes, particularly in resource-limited EDs.

## Data Availability

The data from this study are not publicly archived but may be made available by the authors upon reasonable request, subject to the approval of the Institutional Review Board of the Chang Gung Medical Foundation.

## Abbreviations

ED: Emergency Department
OHCA: Out-of-Hospital Cardiac Arrest
ICU: Intensive Care Unit
GCS: Glasgow Coma Scale
AOR: Adjusted Odds Ratio
CI: Confidence Interval
AIC: Akaike Information Criterion
STROBE: Strengthening the Reporting of Observational Studies in Epidemiology
